# 31. Stepwise Expansion of Antimicrobial Stewardship Program and Its Impact on Antibiotic Use and Resistance Rates in a Tertiary Care Hospital in Korea

**DOI:** 10.1093/ofid/ofab466.233

**Published:** 2021-12-04

**Authors:** Dong Hoon Shin, Hyung-sook kim, Eunjeong Heo, Myoung Jin Shin, Nak-Hyun Kim, Hyunju Lee, Jeong Su Park, Kyoung Un Park, Jongtak Jung, Kyoung-Ho Song, Minsun Kang, Jaehun Jung, Eu Suk Kim, Hong Bin Kim

**Affiliations:** 1 Department of Internal Medicine, Seoul National University College of Medicine, Seoul, Korea, Seoul, Seoul-t’ukpyolsi, Republic of Korea; 2 Seoul National University Bundang Hospital, Seoungnam-si, Kyonggi-do, Republic of Korea; 4 Department of Pediatrics, Seoul National University Bundang Hospital, Seongnam, Korea, Seongnam, Kyonggi-do, Republic of Korea; 5 Department of Laboratory Medicine, Seoul National University Bundang Hospital, Seongnam, Korea, Kyonggi-do, Kyonggi-do, Republic of Korea; 6 Gil Medical Center, Kyonggi-do, Kyonggi-do, Republic of Korea

## Abstract

**Background:**

The U.S. Centers for Disease Control and Prevention released the core elements of antimicrobial stewardship program (ASP). In some countries, however, they may be difficult to apply in countries with limited resources. In this study, we evaluated the impact of successful ASP implementation on antibiotic use and resistance rates in an institution with limited infrastructural support.

**Methods:**

A series of ASP activities were reviewed according to the core elements of ASP. The retrospective data of all hospitalized patients at a tertiary care teaching hospital was collected from January 2010 to December 2019, including antibiotic prescription data and culture results of all clinical specimens. The trends of the antibiotic-resistant rates were compared with nationwide data in Korea. The trend analyses were performed with 2-sided correlated seasonal Mann-Kendall nonparametric tests.

**Results:**

The ASP activities over the past decade were summarized in Table. After activities such as preauthorization were achieved, other ASP activities were added one by one. Also, the infectious disease pharmacists, as ASP co-leaders, mainly carried out the following activities: reducing redundant anti-anaerobic antimicrobials and intravenous fluoroquinolones, and advised the physicians to discontinue the antibiotic prescription in cases when the intervention was plausible. After the ASP implementation, total antibacterial use significantly decreased (*P* < 0.01; Figure). The use of glycopeptides (*P* < 0.01) and fluoroquinolones (*P* < 0.01) gradually decreased, while the use of third-generation cephalosporines did not significantly change (*P*=0.48). There was no significant change in total carbapenems use, but ertapenem use increased (*P*=0.02). Compared with the nationwide data, methicillin-resistant *Staphylococcus aureus* was on a decreasing trend consistently. Although third-generation cephalosporin-resistant *Escherichia coli* increased, third-generation cephalosporin resistant-*Klebsiella pneumoniae* and carbapenem resistant-*Pseudomonas aeruginosa* did not increase.

Table. Antimicrobial stewardship activities for hospitalized patients over the past decade in Seoul National University Bundang Hospital. ASP: antimicrobial stewardship; ID: infectious disease; CDSS: Clinical decision support system

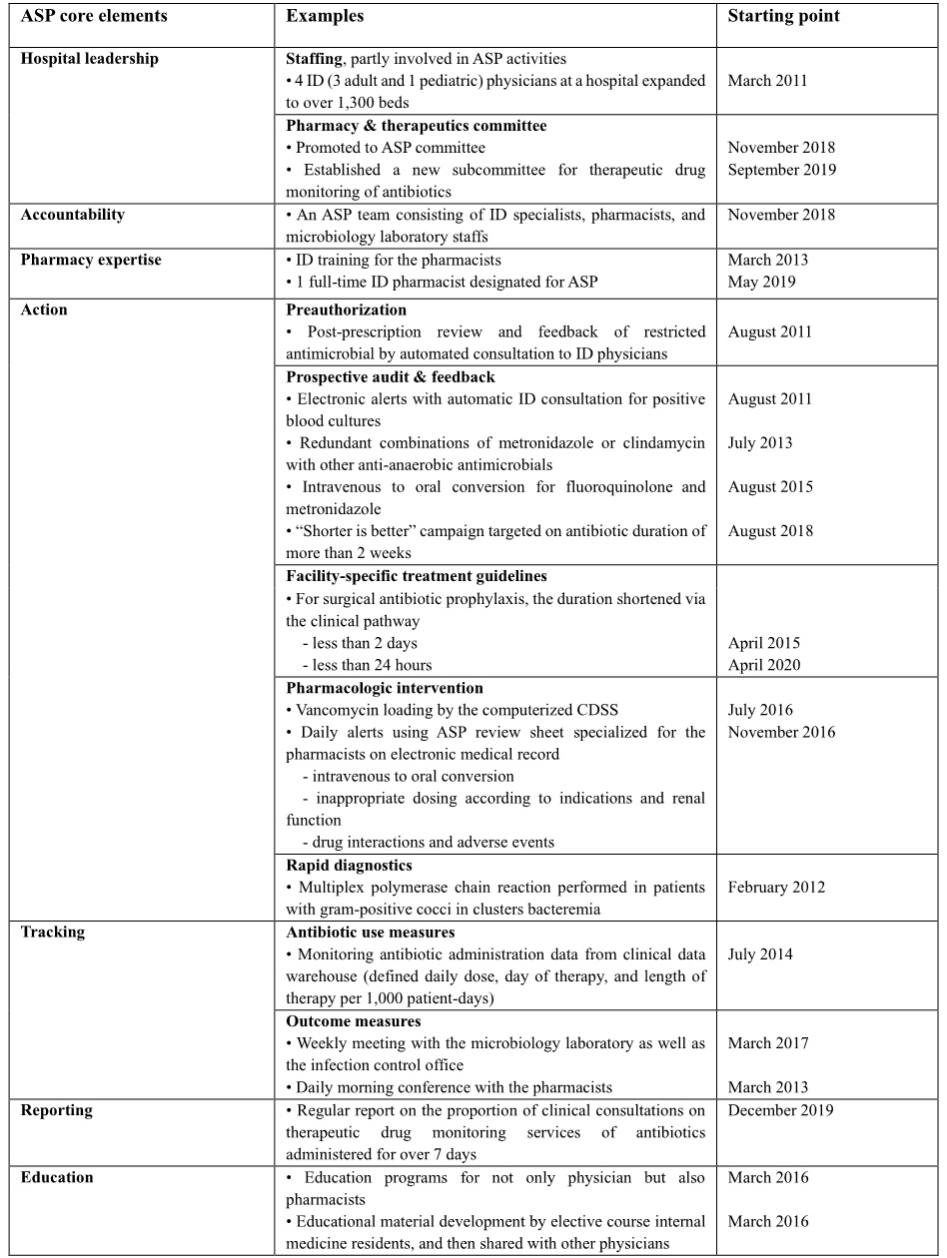

Figure. DOT per 1,000 patient-days in Seoul National University Bundang Hospital and implemented actions of antimicrobial stewardship program. DOT: days of therapy; ID: infectious disease; PCR: polymerase chain reaction; GPC: gram positive cocci; Group 1 carbapenem: ertapenem

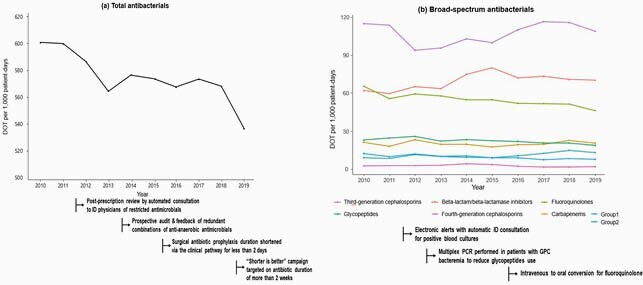

**Conclusion:**

A stepwise implementation of the core ASP elements was effective in improving the appropriate use of antibiotics and reducing the antibiotic resistant organisms, even with limited human resources.

**Disclosures:**

**All Authors**: No reported disclosures

